# Variability in Sperm DNA Fragmentation in Men with Mild/Unexplained Subfertility in a Prospective Longitudinal Intrauterine Insemination Trial

**DOI:** 10.3390/life12111826

**Published:** 2022-11-09

**Authors:** Usha Punjabi, Ella Roelant, Kris Peeters, Ilse Goovaerts, Helga Van Mulders, Diane De Neubourg

**Affiliations:** 1 Centre for Reproductive Medicine, Antwerp University Hospital, 2650 Edegem, Belgium; 2 Department of Reproductive Medicine, Antwerp Surgical Training, Anatomy and Research Centre (ASTARC), Faculty of Medicine and Health Sciences, University of Antwerp, 2000 Antwerpen, Belgium; 3 Clinical Trial Centre (CTC), CRC Antwerp, Antwerp University Hospital, University of Antwerp, Drie Eikenstraat 655, 2650 Edegem, Belgium

**Keywords:** sperm DNA fragmentation, intrauterine insemination, density gradient centrifugation, male infertility

## Abstract

The biological variability of semen and sperm DNA fragmentation (SDF) parameters in a longitudinal intrauterine insemination (IUI) trial over multiple IUI cycles was investigated. A TUNEL assay was used for SDF testing, both before and after density gradient centrifugation. A significant age effect was observed: while semen parameters deteriorated with advancing age, on average, higher SDF values were observed for older males. There was quite some variability observed for both semen and SDF variables. Using fertile threshold values, three patient categories were distinguished: those with a high SDF in all samples, those with low SDF in all samples and those who fluctuated between high and low during the whole IUI trial. Density gradient centrifugation increases SDF. However, the three patient categories react differently after semen processing. A large percentage of those with high SDF retain their high SDF even after gradient centrifugation. The SDF fluctuaters react with a high SDF after gradient centrifugation. The low SDF category, on the contrary, distributes itself evenly between the three categories after gradient centrifugation. SDF testing after semen processing might be indispensable for therapeutic purposes, probably influencing medical decision-making. In order to isolate fluctuaters, a second SDF testing might be advocated in certain cases. SDF after semen processing is indispensable for therapeutic management.

## 1. Introduction

Although semen analysis remains the cornerstone for male factor infertility diagnosis, technical variations and biological variability (within and between individuals) makes the correct interpretation of laboratory results difficult [1,2]. In the last decade, sperm DNA fragmentation (SDF) testing has been indicated in male subfertility diagnosis [3,4]. Several tests, such as the Sperm Chromatin Structure Assay (SCSA) [5], sperm chromatin dispersion test (SCD) [6], TUNEL [7] and the Comet assay [8], have been proposed to detect SDF. However, these tests assess different types of damage and many of them lack standardization. This hampers the clinical use of the reported threshold values for counselling and treatment of men with infertility [9,10]. 

SDF is higher in subfertile men [11,12] as compared to the fertile group and only partially related to semen quality [13,14]. Environmental, technical and biological factors are implicated in the substantial intra-individual variability observed in routine semen parameters [1,15]. Variations in SDF testing, using the SCSA test, were found to be ‘low’ to ‘significant’ by several authors [16,17,18,19], leading to different recommendations for a single or repeated testing. With TUNEL assay, a retest was recommended with higher values [20] or after longer periods of abstinence [21]. As a result, most laboratories undertake SDF testing on one semen sample, assuming that results from a single measurement represent the patient’s condition and is both stable and associated with a good diagnostic value [22]. 

Intrauterine insemination (IUI) and assisted reproductive treatments (ART) such as in vitro fertilization (IVF) or intracytoplasmic sperm injection (ICSI) involve participation in one or more cycles preceded by a diagnostic semen analyses. Investigations undertaken to verify the stability of semen parameters and SDF in a longitudinal ART protocol are lacking.

The objective of this study was to evaluate the degree of SDF variations in an IUI-trial over a period of 3 years.

## 2. Materials and Methods

### 2.1. Study Design and Participants

This was a monocentric, prospective cohort study conducted at the Centre for Reproductive Medicine of the Antwerp University Hospital, Belgium (clinicaltrials.gov NCT03319654). During the period of October 2017 until October 2020, couples who were about to initiate a natural cycle IUI treatment protocol after an infertility duration of 12 months, at minimum, were invited to join the study. Women with ovulation disorders, endocrine disorders (polycystic ovarian syndrome, abnormal thyroid function and prolactin levels, and hypogonadotropic hypogonadism), prior ovarian surgery, prior IUI cycles, moderate to severe endometriosis and double-sided tubal disease were excluded. Men with a severe male factor (initial sperm count <5 million/mL) were also excluded from the study.

Couples were classified as having unexplained subfertility when the fertility investigations showed at least one patent fallopian tube, a regular ovulatory menstrual cycle and a normal diagnostic semen analysis. Mild male infertility was defined as one or more abnormal diagnostic semen parameters with a pre-wash total progressive motile sperm count (TPMC) above 5 million according to the WHO criteria [23]. The couples underwent natural cycle IUI until pregnancy was achieved for a maximum of four cycles.

The Ethical Committee of the Antwerp University hospital and the University of Antwerp approved the study protocol (Belgian registration number: B300201733352, approved on 11 September 2017). Informed consent to use the sperm rest fractions for DNA fragmentation testing was obtained from all the participants. I patients also gave their consent to use their clinical data for research purposes. 

### 2.2. Semen Analysis and Semen Processing

Patients were instructed to maintain 2–7 days of sexual abstinence. All semen samples were collected at the laboratory, and any ejaculate fraction missing was reported. Samples were weighed and an analysis was initiated within 60 min after ejaculation conforming to the international standards of ISO 15189 (International Standards Organization, 2012). Standard semen parameters including sperm concentration and motility were determined using WHO [23] recommendations and complying with the checklist for acceptability reported by Björndahl et al. [9].

All staff members were trained in basic semen analysis (ESHRE—European Society for Human Reproduction and Embryology Basic Semen Analysis Courses) [24,25] and participated regularly in internal and external quality control programs (Institute of Public Health, Belgium and ESHRE External Quality Control Schemes, Sweden) [26]. 

A part of the same semen sample was treated with a two-step discontinuous density gradient [27] using Puresperm^®^ (Nidacon, International AB, Gothenburg, Sweden). Briefly, 40% and 80% Puresperm^®^ (Nidacon, International AB, Gothenburg, Sweden) density gradients were prepared using 1.5 mL of each suspension. Semen (1.0–1.5 mL) was layered on the top of each gradient and centrifuged for 20 min at 300× *g*. After centrifugation of the top seminal plasma layer, the 40% upper layer and the 40–80% interface were discarded and the remaining spermatozoa in the 80% pellet were collected from the bottom of the tube and washed once for 10 min with human tubal fluid (HTF Hepes, Gynotec, Malden, The Netherlands) supplemented with albumin (Human Albumin 20%, CAF-DCF, Brussels, Belgium). 

### 2.3. SDF Testing

Assessment of SDF was performed using the TUNEL assay described by Mitchell et al. [28]. Briefly, spermatozoa were incubated for 30 min at 37 °C with LIVE/DEAD^®^ Fixable Dead Cell Stain (far red) (Molecular Probes, Life technologies, Eugene, OR, USA), after which the cells were washed 2× with phosphate buffered saline (PBS, GIBCO Life technologies, Paisley, UK) before being incubated with 2 mM dithiothreitol (DTT, Sigma-Aldrich, Overijse, Belgium) for 45 min, following which the samples were washed 2× in PBS and fixed in 3.7% formaldehyde (Sigma-Aldrich, Overijse, Belgium) for 20 min at 4 °C. As we have previously shown that storage of the sample at 4 °C affects reproducibility [14], the assay was carried out directly after fixation without storage in 0.1 M glycine. For the assay, the spermatozoa were washed 2× and centrifuged before being resuspended in 500 µL of fresh permeabilization solution (100 mg Sodium citrate, 100 µL Triton X–100 in 100 mL dH_2_O) and incubated for 5 min at 4 °C. The cells were washed 2× with PBS. The assay was performed using the fluorescein In Situ Cell Death Detection Kit (Roche Diagnostics, Mannheim, Germany) using Accuri C6 flow cytometer (BD Sciences, Erembodegem, Belgium). For each sample, 5000–10,000 events were recorded at a flow rate of 35 µL/min. The positive control samples were treated with 5 µL of DNase I (Qiagen, Hilden, Germany) 1500 Kunitz Units for 30 min at room temperature. For a negative control, all the components of the labelling solution except the enzyme terminal deoxynucleotidyl transferase was included. 

The test was conducted on both the neat semen (ejaculate-E) and after density gradient centrifugation (gradient-G), and the results presented as follows:

SDF total: percentage of the entire sperm population that was positive for DNA fragmentation.

SDF vital: percentage of the sperm population that were alive and positive for DNA fragmentation.

### 2.4. Intrauterine Insemination

On the day of insemination, the semen sample obtained was analyzed and processed by density gradient centrifugation. In case the semen sample was deemed sufficient for IUI with an inseminating progressive motile count (IPMC) of ≥2 million [29], the sperm rest fraction was used for SDF testing. The prepared sample was kept at room temperature and insemination was carried out using a soft IUI catheter (Wallace^®^ Intrauterine Insemination Catheters, Cooper Surgical, The Hague, The Netherlands) rinsed with HTF and albumin. The inseminating volume was held constant between 0.3 and 0.5 mL [30].

### 2.5. Treatment Outcome

A clinical pregnancy with fetal heartbeat was diagnosed by ultrasonography. Live births were registered in case of a birth occurring after 22 completed weeks of gestational age with evidence of life. 

### 2.6. Statistical Analysis

Descriptive statistics (mean, standard deviation (SD), median, interquartile range (IQR) and range) are reported for the patient characteristics, semen parameters and SDF in the diagnosis and the IUI samples. Spearman correlation was calculated between SDF total-E (SDF vital-E, respectively) and patient characteristics and semen parameters in the diagnosis sample. 

To estimate the intraclass correlation (ICC) and the intra- and inter-variability of the SDF variables and semen parameters, a linear mixed model was fitted with these variables as the outcome, male age as a fixed effect and patient as a random effect to correct for samples coming from the same patient. In this analysis, all samples are used (diagnosis and IUI). To have assumptions of normality and homoscedasticity better satisfied, all outcomes except progressive motility were log transformed. A Bland–Altman plot comparing TPMC (SDF total-E, respectively) between diagnosis and first IUI cycle split up according to the time interval between the measurements (> or ≤90 days) is shown. 

The following threshold values obtained from fertile men and sperm donors with proven fertility were used [12]: 

SDF total in ejaculate (SDF total-E): ≤13%; 

SDF vital in ejaculate (SDF vital-E): ≤2%; 

SDF total in gradient (SDF total-G): ≤8%; 

SDF vital in gradient (SDF vital-G): ≤1%. 

The experimental results obtained led to three distinct categories: ‘Low’ SDF in all available samples, ‘High’ SDF in all available samples and ‘Fluctuaters’ who swing between the high and the low conditions. The distribution of these categories for the different SDF variables are given. The number (%) of livebirths in these categories are reported and compared with a Chi-square test or Fisher’s exact test as appropriate. 

Finally, a linear mixed model is fitted with the log of TPMC as outcome and a categorical variable as fixed effect indicating the SDF total-E category that the man belonged to Low/Fluctuater/High and patient as random effect. In this model, male age was also taken as a fixed effect. The same model was also fitted with the SDF total-G categories. In the case of a significant SDF category effect, categories were compared two by two using a Tukey correction for multiple testing.

Analyses were conducted using R 4.1.0.

## 3. Results

### 3.1. Participants

SDF was assessed in 313 semen samples from 112 men (mean number of samples 2.8, SD 1.1) and the number of samples analyzed per patient varying between one and five. In 18 men (16.1%) only one semen sample; in 30 (26.8%), two semen samples; in 23 (20.5%), three semen samples; in 39 (34.8%), four samples; and in 2 (1.8%), five samples were analyzed. All semen samples were treated with density gradient, except for one. In 104 (92.9%) men, a diagnostic semen analyses was performed, together with SDF both before and after density gradient.

### 3.2. Pregnancy Outcome

Mean female age included was 30.4 years (SD = 3.7, range 24.–40.0), and male age was 33 (SD = 5.2, range 21.0–49.0) years. Of the 112 couples included, 26 (23.2%) clinical pregnancies were obtained with 21 (18.8%) live births. Basic descriptive statistics for patient characteristics, semen parameters and SDF in the diagnosis and all IUI samples are presented in Table 1.

### 3.3. Parameters Influencing Biological Variability

Correlation between SDF and other patient characteristics and semen variables show that male age (r = 0.24, *p* = 0.014) and progressive motility (r = −0.25, *p* = 0.010) were weakly correlated with SDF total-E (Table 2). 

**Figure 1:**
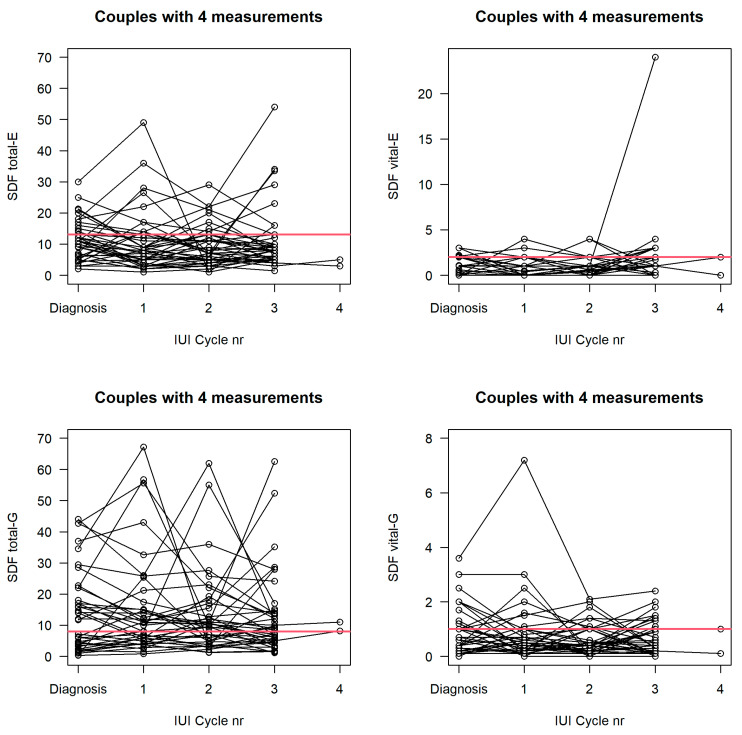
Variations in SDF parameters during the different IUI cycles. SDF = sperm DNA fragmentation; IUI = intrauterine insemination; E = ejaculate; G = gradient.

We calculated the ICC using a linear mixed model where we included male age as a fixed effect. A significant age effect was observed for SDF total-E and SDF total-G with a positive coefficient (estimate on log scale); thus, on average, higher SDF values were found for older males and lower values were found for younger males. For the semen parameters, we observed a negative coefficient, denoting that semen parameters deteriorate with advancing age. The ICC values in the table are quite low in most cases, meaning that there is quite some variation within a person (Figure 1). Intra-individual variability was higher than inter-individual variability for SDF total and SDF vital. The within and between subject variability in sperm concentration and motility was similar, although high (Table 3).

**Figure 2:**
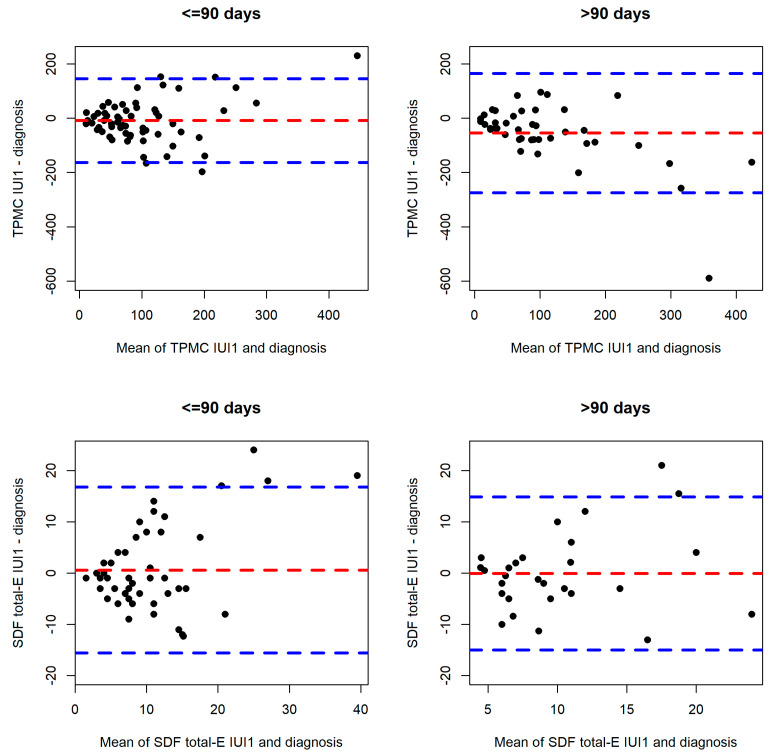
Bland–Altman plots for TMPC and SDF total-E between diagnosis and the first IUI. SDF = sperm DNA fragmentation; IUI = intrauterine insemination; TPMC = total progressive motile count; E = ejaculate; G = gradient.

The different IUI treatments were extended far beyond 3 months of diagnosis, a period typically representing one spermatogenic cycle. Mean time interval between two samples was 80 days (SD, 94; range 6–642 days). Bland and Altman plots for SDF reveal no difference as far as time interval is concerned (Figure 2), but TMPC in ejaculate reveals significant difference after >90 days.

### 3.4. SDF Variability Influencing IUI Outcome

**Figure 3:**
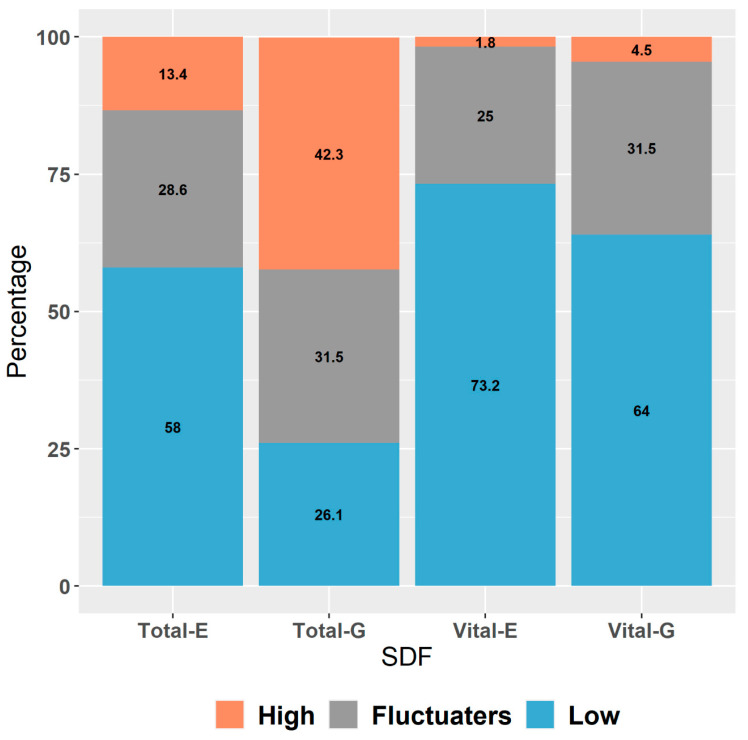
SDF categories in the IUI trial. SDF = sperm DNA fragmentation; IUI = intrauterine insemination; E = ejaculate; G = gradient.

Using fertile threshold values, we distinguished three patient categories (Figure 3): those with a high SDF total-E (13.4%) in all samples, those with low SDF total-E (58.0%) in all samples and those who fluctuated (28.6%) between the two during the whole IUI trial. 

We observed that 66.7% of those with high SDF total-E retain their high SDF total even after the gradient. The SDF total-E fluctuaters (56.3%) react with a high SDF total after the gradient (Table 4). The low SDF total-E category, on the contrary, distributes itself evenly between the three categories after gradient centrifugation. The vital fraction remains more or less stable after preparation.

When considering the potential effect of the different SDF categories on IUI outcome, we observed no significant difference in live births between these three categories (Table 5).

In a linear mixed model, with log TPMC as outcome and with male age and different SDF total-E categories as fixed effects, there is no significant effect of the SDF total-E categories (*p* = 0.775).

Applying the same model but now using the categories after density gradient centrifugation, there is a significant effect of the SDF total-G categories (*p* = 0.001). Comparing the categories (with Tukey correction), we observed a significance between the high SDF and the fluctuaters (*p* = 0.003) and between the high SDF and the low SDF groups (*p* = 0.003). The geometric mean in the fluctuaters (1.68, 95% CI [1.16, 2.42]) is twice as high as in the high SDF group and the same is observed in the low SDF group as compared to the high SDF (1.75, 95% CI [1.18, 2.61]). This trend is also observed in the live births (Table 4), where the live birth rate was twice as high in the low versus the high SDF total-G category (Chi-square test comparing the two groups *p* = 0.105). On the contrary, there was no significance between the low SDF and fluctuaters (*p* = 0.959).

## 4. Discussion

To our knowledge, this is the first prospective study evaluating the variability in SDF measurements through a TUNEL assay, in a longitudinal IUI trial. In an attempt to determine where in the patients clinical IUI pathway SDF testing fits, analyses were carried out both before and after semen processing. 

Variability exists in semen parameters within and among individuals, without clear etiologies for the changes [31]. SDF seems to have a lower biological intra-individual variation than conventional semen parameters [16,17,32]. In one report [32], the DNA fragmentation index (DFI) coefficient of variation (CV) measured via SCSA was significantly lower (9.2%) than sperm count (43.0%), progressive motility (28.3%) and sperm morphology (28.3%). The inter-individual variation is much larger and more evident in the patient group (29.48%) than in sperm donors (17.56%) [32]. Notwithstanding, a study involving 282 infertile men undergoing IUI or ART revealed that the mean CV of DFI was 29%, with about one-third of the patients crossing the threshold levels in repeated analyses [18]. Our study confirms the observation that there is quite some variation in SDF parameters even within individuals with mild/unexplained male infertility and together with the high and low SDF groups; we identify one out of three men who fluctuate between the two. Smits et al. [32] hypothesized that in patients with a more severe disturbance in spermatogenesis, the chromatin structure is so compromised that the deleterious effect of unknown factors causing fluctuations in sperm DNA damage may not be profound as in men with normal spermatogenesis and consequent normozoospermia and normal endocrine levels.

SDF variability is mainly due to genetics, the environmental, abstinence, life style conditions, disease progression or interventional therapy [1,15,33,34,35,36,37]. In our study, BMI, alcohol intake, smoking and abstinence were not related to the variability in SDF results. Male age, on the contrary, significantly influenced both intra- and inter-individual variability, confirming our previous observations that age affects SDF total-E but not SDF vital-E [14]. Germ cell apoptosis during spermatogenesis, which is a normal event, may be less effective in older men resulting in the release of more DNA-damaged sperm [38]. Excessive stress may also be responsible for an increase in DNA damage seen with age [39,40], although results have been controversial [41,42].

According to the group of Muratori [21], the longer the time between the two tests, the greater the intra-individual variation in SDF. Over a period of ~100 days, all the CV’s (*n* = 25) were <20% (one outlier not included), whereas for longer times, the SDF CVs (*n* = 45) were ≥20% in 16 out of 45 patients and lower in 29 patients [21]. We could not confirm this observation. As SDF is weakly correlated with semen parameters, it may be considered as an independent attribute of semen quality for all infertility patients detecting problems not revealed by semen analysis alone. 

The reliability of SDF testing has possible clinical implications for therapeutic management. In addition to intra-individual variability, it is crucial to know how often repeated test results cross the threshold levels that discriminate normal and pathological categories [37]. Alvarez et al. [1] reported that about 5% of infertile men have significant unexplained fluctuations in SCSA parameters. Smit et al. [32] found a >10% CV for the DFI parameter in 47% of patients. Using the SCD test, Esteves et al. [37] defined three categorical classes created on the basis of pre-defined SDF levels: normal (<20%), intermediate (20–29%) and high (>30%). The authors observed a discrepancy rate of approximately 20% in the ejaculates classified as normal or high SDF levels and 40% among patients with intermediate SDF levels. The reason for this discrepancy was unknown and, according to the authors, could be related to the narrower SCD window (20–29%) than in the high and normal categories. 

In order to isolate ‘fluctuaters’, a second SDF testing might be advocated. Since the TUNEL assay is tedious and highly expensive, based on our observations, we could propose to stratify patients at high risk of variability by repeating analyses when:

➢ SDF is ‘high’ in the ejaculate or
➢ SDF is ‘low’ in the ejaculate but, ‘high’ after the gradient

However, our results need to be substantiated first. Sergerie et al. [20] calculated the number of tests needed to estimate the homeostatic setting point for SDF and reported that at 80% confidence, three semen samples are required to obtain an estimate within ±20% of the true setting point.

Sperm preparation via density gradient centrifugation not only result in the isolation of spermatozoa with improved morphology and motility [27,43] but also effectively separating leukocytes and immature germ cells, thereby reducing oxidative stress associated with sperm preparation [44]. We substantiated our previous observations that semen processing via gradient centrifugation increases SDF total-G, but not SDF vital-G [14], probably due to the capacity of density gradient centrifugation to blunt the amount of immature germ cells and leukocytes, thereby reducing oxidative damage in the vital fraction. Defects in sperm chromatin maturation lower sperm defenses towards oxidative attack and/or too high levels of reactive oxygen species in the ejaculate may result in an increased sensitivity of the semen to corruptive agents [45]. However, the group of patients with high, low and fluctuating SDF levels react differently after semen processing. Although not significant, this was also reflected in the number of live births, being twice as high in the low SDF total-G than in the high SDF total-G group, which pleads for the fact that SDF after density gradient centrifugation is indispensable. 

The strengths of the study lies in the methodology. However, the limited study population is a limitation. Although IUI reimbursement in Belgium takes into account female age (until 43 years of age), the patient age was limited to ≤40 years. Including women above 37 years may introduce bias in the results since the outcome measure is live births. This should be taken into consideration when substantiating our results.

Finally, this article highlights the prevalence of variability of SDF in a prospective, longitudinal IUI trial. One out of three men fluctuate between the high and the low SDF groups. Repeat analysis in certain cases could help identify this group. The significance of detecting DNA damage both before and after density gradient centrifugation might be indispensable for therapeutic purposes as the high, low and the fluctuating SDF groups react differently, which alerts us to avoid such damage in the name of ‘best practice’. However, the results need to be substantiated and opportunities created to explore populations with an extreme male factor and the clinical implications in different ART. 

## Figures and Tables

**Table 1:** Basic descriptive statistics for patient characteristics, semen and SDF parameters in the diagnosis and all IUI samples.

Variables/Parameters (n)	Mean (SD)	Median (IQR)	Range
**Male variables:**			
Age, years (112)	33.0 (5.2)	32.5 (7.0)	21.0–49.0
BMI, kg/m^2^ (85)	25.3 (3.5)	24.7 (4.1)	18.4–36.1
Smokers, cigarettes/day (110)	2.4 (5.4)	0.0 (0.0)	0.0–20.0
**Diagnostic variables:**			
Semen parameters:			
Abstinence, days (99)	3.9 (1.6)	4.0 (2.0)	1–7
Sperm concentration, M/mL (104)	66.4 (67.9)	44.2 (58.2)	6.8–512.5
Total sperm count, M (104)	214.3 (197.1)	153.6 (183.1)	14.6–1332.5
Progressive motility, % (104)	52.7 (12.9)	54.0 (16.2)	3–89
Total progressive motile count, M (104)	116.7 (107.4)	85.7 (107.1)	0.4–652.9
Sperm morphology, % (101)	5.2 (3.4)	4.0 (4.0)	0–15
SDF parameters:	10.5 (7.2)		
SDF total in ejaculate, % (104)	1.1 (1.1)	9.0 (7.2)	1.0–42.3
SDF vital in ejaculate, % (104)	13.9 (11.9)	1.0 (0.9)	0–7.0
SDF total after gradient, % (102)	0.8 (0.7)	9.6 (15.5)	0.3–54.6
SDF vital after gradient, % (102)		0.6 (0.8)	0–3.6
**IUI cycle variables:**	4.4 (2.1)		1–14
Semen parameters:	59.4 (38.9)	4.0 (2.0)	6.1–225.0
Abstinence, days (211)	220 (163.4)	50.9 (50.1)	22.4–1365.0
Sperm concentration, M/mL (211)	47.0 (13.3)	178.9 (153.7)	14–96
Total sperm count, M (211)	108.0 (92.4)	46.0 (18.0)	6.3–587.0
Progressive motility, % (211)	6.0 (3.3)	79.9 (92.5)	0.5–17.2
Total progressive motile count, M (211)	9.9 (8.4)	5.3 (4.5)	
Density gradient parameters:	1.4 (2.2)	8.0 (7.0)	1.0–54.0
Inseminating progressive motile count, M (211)	12.1 (12.7)	1.0 (2.0)	0–24.0
SDF parameters:	0.6 (0.8)	8.3 (10.0)	0.8–67.2
SDF total ejaculate, % (209)		0.4 (0.8)	0–7.2
SDF vital ejaculate, % (209)			
SDF total gradient, % (210)			
SDF vital gradient, % (210)			

n = number of observations; M = million; SD = standard deviation; IQR = interquartile range; BMI = body mass index; SDF = sperm DNA fragmentation.

**Table 2:** Spearman correlation between SDF in ejaculate and patient characteristics and semen parameters in the diagnosis sample.

	SDF Total-E (*p* Value)	SDF Vital-E (*p* Value)
Male age	0.24 (**0.014**)	0.11 (0.259)
Body mass index	0.06 (0.597)	−0.07 (0.525)
Alcohol (units/week)	−0.05 (0.614)	−0.06 (0.543)
Smoking (cigarettes/day)	−0.15 (0.132)	−0.06 (0.547)
Abstinence	0.04 (0.721)	−0.06 (0.562)
Volume	−0.08 (0.427)	−0.14 (0.165)
Concentration	0.01 (0.917)	0.13 (0.202)
Total sperm count	−0.03 (0.739)	0.05 (0.603)
Progressive motility	−0.25 (**0.010**)	−0.02 (0.856)
Total progressive motile count	−0.10 (0.325)	0.03 (0.799)
Morphology	0.04 (0.675)	−0.06 (0.520)

E = ejaculate; SDF = sperm DNA fragmentation.

**Table 3:** Results of linear mixed model with SDF and semen parameters as outcome and male age as fixed effect.

Variables	*p* Value Male Age	ICC	Between SD	Within SD	Estimate Age Effect on Log Scale [95% CI]	Estimate Age Effect Back-Transformed [95% CI]
SDF total-E	**0.020**	0.33	0.42	0.60	0.025 [0.004, 0.046]	1.025 [1.004, 1.047]
SDF vital-E	0.952	0.06	0.37	1.49	0.001 [−0.035, 0.038]	1.001 [0.965, 1.038]
SDF total-G	**0.002**	0.54	0.70	0.64	0.047 [0.017, 0.077]	1.048 [1.018, 1.08]
SDF vital-G	0.572	0.13	0.40	1.03	0.008 [−0.02, 0.037]	1.008 [0.98, 1.037]
Concentration	0.400	0.62	0.60	0.47	−0.011 [−0.035, 0.014]	0.989 [0.965, 1.014]
Total sperm count	0.408	0.50	0.54	0.54	−0.010 [−0.033, 0.014]	0.990 [0.967, 1.014]
Progressive motility	0.368	0.42	8.71	10.22	−0.185 [−0.584, 0.215]	
TMPC	0.325	0.58	0.70	0.60	−0.015 [−0.044, 0.015]	0.985 [0.957, 1.015]

All variables are log transformed except progressive motility. IUI = intrauterine insemination; E = ejaculate; G = gradient; TMPC = total progressive motile count; ICC = intraclass coefficient; SD = standard deviation; CI = confidence interval.

**Table 4:** Cross tabulation of SDF total-E category versus SDF total-G category.

	SDF Total-G			
	High	Fluctuaters	Low	Total
**SDF total-E**				
High	10 (66.7%)	3 (20.0%)	2 (13.3%)	15
Fluctuaters	18 (56.3%)	11 (34.4%)	3 (9.4%)	32
Low	19 (29.7%)	21 (32.8%)	24 (37.5%)	64
Total	47 (42.3%)	35 (31.5%)	29 (26.1%)	111

SDF = sperm DNA fragmentation; E = ejaculate; G = gradient.

**Table 5:** Live births per SDF category.

SDF Parameters	No. of Couples (% within 112)	Live Births (% within SDF Category)	*p* Value
**SDF total-E**			
High (>13%)	15 (13.4%)	1 (6.7%)	
Fluctuaters	32 (28.6%)	6 (18.8%)	0.472 (C)
Low (≤13%)	65 (58.0%)	13 (20.0%)	
**SDF vital-E**			
High (>2%)	2 (1.8%)	1 (50.0%)	
Fluctuaters	28 (25.0%)	4 (14.3%)	0.363 (F)
Low (≤2%)	82 (73.2%)	15 (18.3%)	
**SDF total-G**			
High (>8%)	47 (42.3%)	6 (12.8%)	
Fluctuaters	35 (31.5%)	7 (20.0%)	0.272 (C)
Low (≤8%)	29 (26.1%)	8 (27.6%)	
**SDF vital-G**			
High (>1%)	5 (4.5%)	1 (20.0%)	
Fluctuaters	35 (31.5%)	8 (22.9%)	0.762 (F)
Low (≤1%)	71 (64.0%)	12 (16.9%)	

SDF = sperm DNA fragmentation; E = ejaculate; G = gradient; C = Chi square; F = Fisher exact test.

## Data Availability

The data presented in this study are available from the corresponding author upon reasonable request.

## References

[1] Alvarez C., Castilla J.A., Martínez L., Ramírez J.P., Vergara F., Gaforio J.J. (2003). Biological variation of seminal parameters in healthy subjects. Hum. Reprod..

[2] Filimberti E., Degl’Innocenti S., Borsotti M., Quercioli M., Piomboni P., Natali I., Fino M.G., Caglieresi C., Criscuoli L., Gandini L. (2013). High variability in results of semen analysis in andrology laboratories in Tuscany (Italy): The experience of an external quality control (EQC) programme. Andrology.

[3] Aitken R.J., De Iuliis G.N., McLachlan R.I. (2009). Biological and clinical significance of DNA damage in the male germ line. Int. J. Androl..

[4] Barratt C.L., Aitken R.J., Björndahl L., Carrell D.T., de Boer P., Kvist U., Lewis S.E., Perreault S.D., Perry M.J., Ramos L. (2010). Sperm DNA: Organization, protection and vulnerability: From basic science to clinical applications—A position report. Hum. Reprod..

[5] Evenson D.P., Kasperson K., Wixon R. (2007). Analysis of sperm DNA fragmentation using flow cytometer and other techniques. Soc. Reprod. Fertil. Suppl..

[6] Muriel L., Garrido N., Fernández J.L., Remohí J., Pellicer A., de los Santos M.J., Meseguer M. (2006). Value of the sperm DNA fragmentation level, measured by the sperm chromatin dispersion (SCD) test, in the IVF and ICSI outcome. Fertil. Steril..

[7] Chohan K.R., Griffin J.T., Lafromboise M., De Jonge C.J., Carell D.T. (2006). Comparison of chromatin assays for DNA fragmentation evaluation in human sperm. J. Androl..

[8] Lewis S.E., Agbaje I.M. (2008). Using the alkaline comet assay in prognostic tests for male infertility and assisted reproductive technology outcomes. Mutagenesis.

[9] Björndahl B., Barratt C.L., Mortimer D., Jouannet P. (2016). ‘How to count sperm properly’: Checklist for acceptability of studies based on human semen analysis. Hum. Reprod..

[10] Aitken R.J., Bakos H.W. (2021). Should we be measuring DNA damage in human spermatozoa? New light on an old question. Hum. Reprod..

[11] Tamburrino L., Marchiani S., Montoya M., Elia Marino F., Natali I., Cambi M., Forti G., Baldi E., Muratori M. (2012). Mechanisms and clinical correlates of sperm DNA damage. Asian J. Androl..

[12] Punjabi U., Van Mulders H., Goovaerts I., Peeters K., Roelant E., De Neubourg D. (2019). DNA fragmentation in concert with the simultaneous assessment of cell viability in a subfertile population: Establishing thresholds of normality both before and after density gradient centrifugation. J. Assist. Reprod. Genet..

[13] Evgeni E., Charalabopoulos K., Asimakopoulos B. (2014). Human sperm DNA fragmentation and its correlation with conventional semen parameters. J. Reprod. Infertil..

[14] Punjabi U., Van Mulders H., Goovaerts I., Peeters K., Clasen K., Janssens P., Zemstova O., De Neubourg D. (2018). Sperm DNA fragmentation in the total and vital fractions before and after density gradient centrifugation: Significance in male fertility diagnosis. Clin. Biochem..

[15] Oshio S., Ashizawa Y., Yotsukura M., Tohyama Y., Iwabuchi M., Adachi Y., Matsuda H., Tomomasa H., Yoshida S., Takeda K. (2004). Individual variation in semen parameters of healthy young volunteers. Arch. Androl..

[16] Evenson D.P., Jost L.K., Baer R.K., Turner T.W., Schrader S.M. (1991). Individuality of DNA denaturation patterns in human sperm as measured by the sperm chromatin structure assay. Reprod. Toxicol..

[17] Zini A., Kamal K., Phang D., Willis J., Jarvi K. (2001). Biologic variability of sperm DNA denaturation in infertile men. Urology.

[18] Erenpreiss J., Bungum M., Spano M., Elzanaty S., Orbidans J., Giwercman A. (2006). Intra-individual variation in sperm chromatin structure assay parameters in men from infertile couples: Clinical implications. Hum. Reprod..

[19] Oleszczuk K., Augustinsson L., Bayat N., Giwercman A., Bungum M. (2013). Prevalence of high DNA fragmentation index in male partners of unexplained infertile couples. Andrology.

[20] Sergerie M., Laforest G., Boulanger K., Bissonnette F., Bleau G. (2005). Longitudinal study of sperm DNA fragmentation as measured by terminal uridine nick end-labelling assay. Hum. Reprod..

[21] Muratori M., Marchiani S., Tamburrino L., Cambi M., Lotti F., Natali I., Filimberti E., Noci I., Forti G., Maggi M. (2015). DNA fragmentation in brighter sperm predicts male fertility independently from age and semen parameters. Fertil. Steril..

[22] Esteves S.C., Zini A., Coward R.M., Evenson D.P., Gosálvez J., Lewis S.E.M., Sharma R., Humaidan P. (2021). Sperm DNA fragmentation testing: Summary evidence and clinical practice recommendations. Andrologia.

[23] World Health Organization (2010). WHO Laboratory Manual for the Examination of Human Semen and Semen-Cervical Mucus Interaction.

[24] Punjabi U., Spiessens C., Ombelet W., Bosmans E., Vandeput H., Vereecken A., Renier M., Hoomans E. (1998). Basic semen analysis courses: Experience in Belgium. Modern ART in the 2000′s—Andrology in the Nineties.

[25] Björndahl L., Barratt C.L., Fraser L.R., Kvist U., Mortimer D. (2002). ESHRE basic semen analysis courses 1995–1999: Immediate beneficial effects of standardized training. Hum. Reprod..

[26] Punjabi U., Wyns C., Mahmoud A., Vernelen K., China B., Verheyen G. (2016). Fifteen years of Belgian experience with external quality assessment of semen analysis. Andrology.

[27] Punjabi U., Gerris J., Van Bijlen J., Delbeke L., Buytaert P. (1990). Comparison between different pre-treatment techniques for sperm recovery prior to IUI-GIFT-IVF. Hum. Reprod..

[28] Mitchell L.A., De Iuliis G.N., Aitken R.J. (2010). The TUNEL assay consistently underestimates DNA damage in human spermatozoa and is influenced by DNA compaction and cell vitality: Development of an improved methodology. Int. J. Androl..

[29] Punjabi U., De Neubourg D., Van Mulders H., Cassauwers W., Peeters K. (2018). Validating semen processing for an intrauterine program should take into consideration the inputs, actions and the outputs of the process. Andrologia.

[30] Punjabi U., Van Mulders H., Van de Velde L., Goovaerts I., Peeters K., Cassauwers W., Lyubetska T., Clasen K., Janssens P., Zemtsova O. (2021). Time intervals between semen production, initiation of analysis, and IUI significantly influence clinical pregnancies and live births. J. Assist. Reprod. Genet..

[31] Mallidis C., Howard E.J., Baker H.W. (1991). Variation of semen quality in normal men. Int. J. Androl..

[32] Smit M., Dohle G.R., Hop W.C., Wildhagen M.F., Weber R.F., Romijn J.C. (2007). Clinical correlates of the biological variation of sperm DNA fragmentation in infertile men attending an andrology outpatient clinic. Int. J. Androl..

[33] Morrison C.D., Brannigan R.E. (2015). Metabolic syndrome and infertility in men. Best Pract. Res. Clin. Obstet. Gynaecol..

[34] Jeng H.A., Pan C.H., Chao M.R., Chiu C.C., Zhou G., Chou C.K., Lin W.Y. (2016). Sperm quality and DNA integrity of coke oven workers exposed to polycyclic aromatic hydrocarbons. Int. J. Occup. Med. Environ. Health.

[35] Agarwal A., Majzoub A., Baskaran S., Panner Selvam M.K., Cho C.L., Henkel R., Finelli R., Leisegang K., Sengupta P., Barbarosie C. (2020). Sperm DNA Fragmentation: A New Guideline for Clinicians. World J. Mens. Health.

[36] Teixeira T.A., Oliveira Y.C., Bernardes F.S., Kallas E.G., Duarte-Neto A.N., Esteves S.C., Drevet J.R., Hallak J. (2021). Viral infections and implications for male reproductive health. Asian J. Androl..

[37] Esteves S.C., López-Fernández C., Martínez M.G., Silva E.A., Gosálvez J. (2022). Reliability of the sperm chromatin dispersion assay to evaluate sperm deoxyribonucleic acid damage in men with infertility. Fertil. Steril..

[38] Print C.G., Loveland K.L. (2000). Germ cell suicide: New insights into apoptosis during Spermatogenesis. Bioassays.

[39] Pasqualotto F.F., Sharma R.K., Potts J.M., Nelson D.R., Thomas A.J., Agarwal A. (2000). Seminal oxidative stress in patients with chronic prostatitis. Urology.

[40] Cocuzza M., Athayde K.S., Agarwal A., Sharma R., Pagani R., Lucon A.M., Srougi M., Hallak J. (2008). Age-related increase of reactive oxygen species in neat semen in healthy fertile men. Urology.

[41] Tirado E. (2012). Concurrent sperm DNA fragmentation and oxidative stress assessment on 2281 male semen samples. Fertil. Steril..

[42] Alshahrani S., Agarwal A., Assidi M., Abuzenadah A.M., Durairajanayagam D., Ayaz A., Sharma R., Sabanegh E. (2014). Infertile men older than 40 years are at higher risk of sperm DNA damage. Reprod. Biol. Endocrinol..

[43] Morrell J.M., Moffatt O., Sakkas D., Manicardi G.C., Bizzaro D., Tomlinson M., Nilsson H., Holmes P.V. (2004). Reduced senescence and retained nuclear DNA integrity in human spermatozoa prepared by density gradient centrifugation. J. Assist. Reprod. Genet..

[44] Aitken R.J., Clarkson J.S. (1988). Significance of reactive oxygen species and antioxidants in defining the efficacy of sperm preparation techniques. J. Androl..

[45] Muratori M., Tarozzi N., Cambi M., Boni L., Iorio A.L., Passaro C., Luppino B., Nadalini M., Marchiani S., Tamburrino L. (2016). Variation of DNA fragmentation levels during density gradient sperm selection for assisted reproductive techniques—A possible new male predictive parameter of pregnancy?. Medicine.

